# Detection of *Burkholderia mallei* in Microbiological Culture: A Comparative Analysis of PCR Primer Sets

**DOI:** 10.3390/pathogens14080766

**Published:** 2025-08-02

**Authors:** Jéssica Cristine K. Moriya, Paula Adas P. Suniga, Ana Clara L. Araújo, Maria Goretti Santos, Juliana S. G. Rieger, Cynthia Mantovani, Rodrigo Jardim, Márcio Roberto Silva, Flábio R. Araújo, Lenita R. Santos

**Affiliations:** 1 CNPq Scholarship/Embrapa Beef Cattle, Campo Grande 79106-550, MS, Brazil; biokuramoto@gmail.com; 2 Embrapa Beef Cattle/Ministry of Agriculture, Livestock and Food Supply Scholarship, Campo Grande 79106-550, MS, Brazil; paula_adas@hotmail.com (P.A.P.S.); juliana_vet11@hotmail.com (J.S.G.R.); cymant@hotmail.com (C.M.); 3 CNPq/PIBIC Program/Embrapa Beef Cattle, Campo Grande 79106-550, MS, Brazil; ana.clara.leonardo@ufms.br; 4 Embrapa Beef Cattle, Campo Grande 79106-550, MS, Brazil; goretti.santos@embrapa.br (M.G.S.); flabio.araujo@embrapa.br (F.R.A.); 5 Oswaldo Cruz Institute, Fiocruz, Rio de Janeiro 21040-900, RJ, Brazil; 6 Embrapa Dairy Cattle, Juiz de Fora 36038-330, MG, Brazil; marciorobertosilva@yahoo.com.br

**Keywords:** glanders, PCR, *Burkholderia mallei*, microbiological culture

## Abstract

Glanders is a highly contagious and often fatal zoonotic disease of equids caused by *Burkholderia mallei*, a pathogen of significant concern due to its potential for bioterrorism. In Brazil, glanders remains endemic, particularly among working equids in the Northeast region. Diagnostic confirmation typically involves serology, culture, and polymerase chain reaction (PCR), although false-negative PCR results have been increasingly reported. This study aimed to evaluate the diagnostic performance and analytical sensitivity of four *B. mallei*-specific PCR primer sets using samples from 30 seropositive equids. Microbiological cultures were obtained from various organs and swabs, followed by PCR targeting four genomic regions: *fliP*-IS407A(a), *fliP*-IS407A(b), Burk457, and Bm17. All animals were confirmed positive for *B. mallei* via culture, but PCR detection rates varied significantly across primer sets. The *fliP*-IS407A(b) primer set showed the highest sensitivity, detecting 86% of samples, while the WOAH-recommended *fliP*-IS407A(a) set had the lowest performance (13.4%). Analytical sensitivity assays confirmed that *fliP*-IS407A(b) and Bm17 primers detected DNA concentrations as low as 0.007 ng, outperforming the others. These findings suggest that certain widely used primer sets may lack sufficient sensitivity for reliable detection of *B. mallei*, especially in chronically infected animals with low bacterial loads. The study underscores the need for ongoing validation of molecular diagnostics to improve the detection and control of glanders in endemic regions.

## 1. Introduction

Glanders is a contagious and often fatal disease caused by the Gram-negative bacterium *Burkholderia mallei*, primarily affecting equids such as horses, mules, and donkeys [1]. It is also a zoonotic disease, with documented human cases dating back to the early 20th century [2,3]. Due to its pathogenicity and potential use as a bioterrorism agent, *B. mallei* is classified by the U.S. Centers for Disease Control and Prevention (CDC) as a Tier 1 select agent [4].

In equids, the clinical presentation varies depending on the host species and the course of infection. In donkeys and mules, the disease often progresses rapidly and is characterized by high fever, nasal swelling, dyspnea, and pneumonia, commonly leading to death within a few days. In contrast, horses tend to experience a chronic form, which may remain subclinical for extended periods [1,5]. During this time, infected animals can intermittently or continuously shed the pathogen, acting as asymptomatic reservoirs and contributing to the spread of the disease [6,7].

In horses, clinical signs include inflammatory pustules and ulcers in the nasal conchae and septum, leading to thick yellow nasal discharge and firm swelling of the submaxillary lymph nodes. These lesions often heal with stellate scars. Pulmonary involvement may be observed as reddish nodules with central necrosis, accompanied by fever, progressive weakness, coughing, dyspnea, and occasionally diarrhea and polyuria. The cutaneous form, known as “farcy,” is marked by the enlargement of lymphatic vessels, the formation of nodular abscesses that rupture to release oily yellow exudate, and the appearance of dry ulcers. Pyogranulomatous nodules may also form in internal organs such as the liver and spleen, contributing to systemic deterioration. Transmission occurs through direct contact with infectious discharges, inhalation, ingestion of contaminated feed or water, or through skin lesions. The incubation period is highly variable, ranging from a few days to several months, which complicates early detection and control measures [1,7].

In Brazil, symptomatic cases of glanders are frequently reported among equids used for labor in the sugarcane fields of the Northeast region, where strenuous workloads and the common practice of housing mules and horses together facilitate disease transmission [8,9,10]. However, both asymptomatic and symptomatic cases have also been reported in other regions of the country [11,12,13,14]. Notably, a recent case of human glanders has also been reported in Brazil [15].

According to the World Organisation for Animal Health (WOAH), serological testing provides supportive evidence of *B. mallei* infection. A complement fixation test (CFT) titer of 1:5 or higher is generally considered indicative of infection. However, positive CFT results should be confirmed with a secondary test of equal or greater sensitivity and specificity. Recommended confirmatory methods include a *B. mallei*-specific lipopolysaccharide (LPS) Western blot, an indirect ELISA targeting a recombinant type VI secretion system protein, or a competitive ELISA (C-ELISA) using *B. mallei*-specific monoclonal antibodies. Despite the utility of serological assays, definitive diagnosis requires the isolation and identification of *B. mallei* from clinical or equine-derived samples, with confirmation via biochemical assays or polymerase chain reaction (PCR) techniques [7].

*Burkholderia mallei* demonstrates robust but slow growth on a variety of culture media, including sheep blood agar. To maximize detection, incubation for at least 72 h is recommended, with glycerol enrichment enhancing growth. On sheep blood agar, *B. mallei* typically forms small, grayish, shiny colonies that may be easily obscured by faster-growing contaminants. As such, cultures should be monitored closely beyond the initial 72 h period. Other recommended media include glycerol potato agar and glycerol broth, where *B. mallei* develops a characteristic slimy pellicle. Growth on nutrient agar is less pronounced, and on gelatin, it is typically inadequate [7].

For the confirmation of presumptive *B. mallei* isolates, the World Organisation for Animal Health (WOAH) recommends the use of biochemical methods and PCR-based assays. However, biochemical characterization presents several important limitations. The in vitro growth characteristics of *B. mallei* can vary, often requiring the use of freshly isolated strains, which reduces diagnostic flexibility. Furthermore, strain-dependent variability in biochemical reactions can lead to inconsistent or inaccurate results [7].

In contrast, PCR offers several advantages for confirming *B. mallei* infection. It delivers high specificity, enabling differentiation from other *Burkholderia* species, and is less time-consuming than biochemical assays. PCR also requires only small amounts of inactivated biological material, reducing the need to handle live cultures and thereby improving biosecurity [16]. It can be performed on both microbiological cultures and directly on tissue samples from suspected cases [11,12,14,16]. By targeting the pathogen’s genetic material, PCR ensures definitive identification and avoids the variability associated with biochemical methods.

Our group conducted a nationwide study to detect *B. mallei* DNA in seropositive equids across all physiographic regions of Brazil. PCR performed on microbiological cultures proved more effective than direct testing of tissue samples, likely due to the low bacterial DNA concentration in tissues, the abundance of host genomic material, and the presence of PCR inhibitors in the tissue matrix [12].

Despite its advantages, PCR also has limitations. Many circulating *B. mallei* strains remain genetically uncharacterized [17], and mutations or recombination events at primer or probe binding sites can result in false-negative results. The WOAH Terrestrial Manual [7] warns that the continued genetic evolution of *B. mallei* could lead to the emergence of variants undetectable by current PCR assays. The pathogen’s genome is highly plastic, undergoing frequent changes driven by insertion sequence (IS)-mediated recombination events [18,19]. Moreover, with few exceptions [17], the performance of various PCR primers has not been systematically evaluated using standardized biological samples, contributing to variability in diagnostic outcomes.

Therefore, this study aimed to compare four PCR primer sets to determine which provides the highest diagnostic sensitivity for detecting *B. mallei* DNA in microbiological cultures. These sets were selected because they represent (i) the current WOAH-recommended standard (*fliP*-IS407A(a)); (ii) an alternative design targeting the same locus but producing a shorter amplicon (*fliP*-IS407A(b)); (iii) a distinct conserved genomic region (Burk457) shown to perform well in recent Brazilian isolates; and (iv) a VNTR locus (Bm17) that offers discriminatory power for differentiating *B. mallei* from closely related species. By directly comparing these primers under standardized conditions, we tested the hypothesis that alternative targets may outperform the current standard, providing evidence-based guidance for more reliable molecular diagnosis of glanders.

## 2. Materials and Methods

### 2.1. Samples

Glanders cases included in this study involved 30 animals—26 horses, 3 mules, and 1 donkey—of both sexes, with or without clinical signs. Diagnosis was based on serological screening using ELISA (Biovetech, Recife, PE, Brazil), followed by confirmatory testing via Western blot (Biovetech, Brazil).

All seropositive animals were euthanized and necropsied, and biological samples were collected and submitted to the BSL-3 Biopec Laboratory at Embrapa Beef Cattle in Campo Grande, Mato Grosso do Sul, Brazil. Serological testing and euthanasia procedures were conducted in accordance with the Brazilian Ministry of Agriculture, Livestock and Food Supply (MAPA) Normative Instruction [20]. Accordingly, no animals were euthanized for experimental purposes.

Samples were collected from various organs and tissues, including the lungs, the left cranial lung lobe, and the accessory lung lobe. Nasal swabs, palate swabs, and a left nostril swab were performed, as well as a frontal sinus swab. In the lymphatic system, submandibular, parotid, mandibular, sublingual, mediastinal, retropharyngeal, and pancreatic lymph nodes were identified, along with pulmonary lymph nodes and other unspecified lymph nodes.

From the digestive system and related organs, samples included the liver, spleen, and kidney. Additionally, purulent abscess content was noted, and samples of subcutaneous fat and heart fat were collected. There were also mentions of unidentified organs, possibly related to the liver.

### 2.2. Microbiological Culture

After decontamination with 70% ethanol for 5 min, the tissue samples were dissected for lesion identification and macerated in liquid brain heart infusion (BHI) medium using a TissueLyser (Quiagen, Venlo, The Netherlands). The resulting supernatants, along with swabs and secretions, were cultured on an agar base supplemented with 5% defibrinated sheep blood and 2% glycerin (blood agar glycerin—BAG), as well as in liquid BHI medium containing 2% glycerin. No antibiotics were added to the media, except for polymyxin B (50 U/mL) and penicillin G (100 U/mL), with cultures maintained under agitation. After 24 h of incubation at 37 °C, the liquid cultures were plated on BAG, and bacterial growth was assessed at 24, 48, and 72 h. The Brazilian reference strain BAC 86/19 (NCBI identifier SAMN28964121) [6] was cultured in parallel with the suspicious samples.

Colonies were classified as *Burkholderia*-compatible if they exhibited characteristic morphology: small, pinpoint, rounded, mucoid, grayish-white colonies with a translucent halo and no hemolysis. These colonies were subcultured for further analysis. We also analyzed the colonies with phenotypic differences to *B. mallei*. Each isolate was subjected to Gram staining and identification using matrix-assisted laser desorption/ionization time-of-flight mass spectrometry (MALDI-TOF).

### 2.3. MALDI-TOF

For MALDI-TOF analysis, bacterial samples were inactivated with absolute ethanol, and protein profiling was performed using the MALDI Biotyper™ system (Bruker Daltonics, Billerica, MA, USA), following established protocols [21,22,23].

Spectra were analyzed using MALDI Biotyper™ software v.3.1 with the MBT Compass Library DB-7311 (v.7.0.0.0), comprising 7311 main spectral profiles (MSPs) from 434 genera and 2509 microbial species. Since this database does not include MSPs for *B. mallei* or *B. pseudomallei*, we supplemented it with reference spectra for *B. mallei* ATCC 23344 and *B. pseudomallei* from the Robert Koch Institute (RKI), Berlin [24], as well as MSPs previously generated by our research group, including *B. mallei* ATCC 15310 and seven clinical isolates of *B. mallei* and *B. pseudomallei* [25].

### 2.4. Inhibition of Contaminants in Microbiological Cultures

Contaminant species identified in the cultures were reviewed in the literature to determine their known antimicrobial susceptibility profiles. These isolates were then tested against a range of antimicrobial concentrations previously reported as effective. The Brazilian reference *B. mallei* strain BAC 86/19 [6] was simultaneously exposed to the same conditions. Based on comparative growth data, specific antimicrobial concentrations were selected to inhibit the contaminants while allowing the selective growth of *B. mallei* BAC 86/19.

Accordingly, the following culture media and antimicrobial agents were employed: BAG medium supplemented with disodium ticarcillin (32 µg/mL), ampicillin (32 µg/mL), and sulfamethoxazole-trimethoprim (50 µg/mL–10 µg/mL). In addition, the semi-selective BM medium described by Kinoshita et al. [26] was utilized, containing cycloheximide (50 µg/mL), disodium ticarcillin (16.7 µg/mL), sodium fosfomycin (197.6 µg/mL), polymyxin B (50 U/mL), and crystal violet (3 mg/L).

### 2.5. PCR

DNA extraction was performed from bacterial culture in BHI broth and bacterial isolates that presented a morphological profile consistent with *B. mallei*, following a modified protocol based on van Embden et al. [27]. To ensure the accuracy of our extraction process, we utilized *Escherichia coli* strain TOP10 (Invitrogen) as a negative control for DNA extraction.

For subsequent PCR analyses, several *B. mallei*-specific genomic regions were targeted. Initially, the primer set described by Scholz et al. [16] was used to amplify a 989 bp fragment within the *fliP*-IS407A region, referred to in this study as *fliP*-IS407A(a). These primers are recommended in the WOAH Terrestrial Manual, Chapter 3.6.10—Glanders and Melioidosis [7].

Additionally, primers based on the work of Abreu et al. [11] were used to amplify a 528 bp fragment from the *fliP*-IS407A region, referred to in this study as *fliP*-IS407A(b). These primers had been previously described: the forward primer by Scholz et al. [16] and the reverse primer by Tomaso et al. [28], together yielding a 528 bp product.

**Figure 1:**
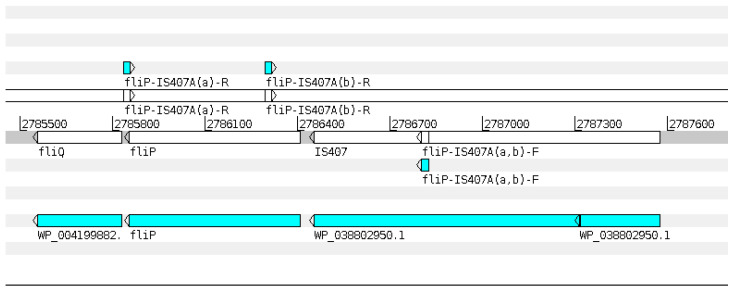
Genomic coordinates targeted by the fliP-IS407A(a) and (b) primers, along with the location of the IS407A insertion site within the *fliP* gene of the *Burkholderia mallei* reference strain ATCC 23344.

The genomic coordinates targeted by the *fliP*_IS407A(a) and (b) primers, as well as the site of gene insertion, are shown in Figure 1.

We also targeted the Burk457 region, which encodes a hypothetical protein, using primers designed by Fonseca Junior et al. [29] to generate a 457 bp fragment.

Finally, the Bm17 primer set, targeting a VNTR locus, was used to yield a 281 bp amplicon for *B. mallei* or a 321 bp amplicon for *B. pseudomallei* [30] (Table 1).

The described primer sets were used to amplify their respective targets in bacterial isolates obtained from clinical samples of equids with positive serology for glanders. In addition, we assessed the analytical sensitivity of each primer set by performing serial dilutions of DNA from the *B. mallei* control strain BAC 86/19. The analysis was conducted by two operators to ensure the reliability of the results obtained.

For the serial dilution of the DNA from the BAC 86/19 strain, we started from an initial concentration of 200 ng/µL down to 0.0003 ng/µL, totaling 20 dilutions. For each PCR, a negative control (nuclease-free water) was included. The reactions followed the specific protocol for each primer evaluated. Subsequently, the amplified products were subjected to agarose gel electrophoresis, allowing for the analysis of DNA detection at different concentrations.

Each PCR included a negative control (nuclease-free water) and a positive control (DNA from *B. mallei*, strain BAC 86/19) [6]. Reactions were prepared in a final volume of 20 μL, using GoTaq Colorless Master Mix (2X) (Promega, Madison, Wisconsin, USA), 0.25 µM of each primer, and 500 ng of template DNA. Amplification was performed using an automated thermocycler (Veriti 96-well Fast Thermal Cycler, Applied Biosystems, Foster City, CA, USA), and PCR products were analyzed by agarose gel electrophoresis. The amplification conditions followed those originally described by the respective authors.

### 2.6. Statistical Analysis

The detection percentage for each individual primer was calculated with a 95% confidence interval (CI) using Epi Info software, version 3.5.8 (2008). To evaluate agreement between two primers in paired samples, the McNemar test—a nonparametric statistical method—was applied using VassarStats (http://vassarstats.net/propcorr.html, accessed on 15 May 2025). Diagnostic performance indicators, including Cohen’s Kappa, sensitivity, specificity, positive predictive value (PPV), and negative predictive value (NPV), were calculated by treating one primer as the reference standard, using OpenEpi (https://www.openepi.com/Menu/OE_Menu.htm, accessed on 15 May 2025). Additionally, the percentage of positive results detected by each primer pair was determined by summing both concordant and discordant positive results between the two primers.

## 3. Results and Discussion

Using all four primer sets, PCR analysis of microbiological cultures successfully identified *B. mallei* in all 30 equids (100%). However, significant differences in detection sensitivity were observed among the primer sets. The fliP-IS407A(b) primer set demonstrated the highest sensitivity, yielding the greatest number of strong positive reactions, followed in descending order by Bm17, Burk457, and fliP-IS407A(a) (Table 2).

Table 3 shows that the two primers, which individually detected the highest number of positive samples, would, when used together, detect 325 out of the 328 positive samples (99.08%).

Regarding the *fliP*-IS407A target, comparative genomic analysis between *Burkholderia pseudomallei* strain K96243 and *B. mallei* strain ATCC 23344 revealed a key structural difference in the *fliP* gene. In *B. pseudomallei*, the *fliP* gene is intact and functional (762 bp), whereas in *B. mallei*, a recombination event disrupts this gene at nucleotide position 235. This disruption involves the insertion of a ~60 kb DNA segment, with the insertion sequence IS407A located immediately upstream of the insertion site—suggesting its role in the recombination. This genomic rearrangement, unique to *B. mallei*, served as the basis for the design of species-specific PCR primers [16]. The primer set developed from this region, referred to in this study as *fliP*-IS407A(a), is currently recommended by the World Organisation for Animal Health [7] in its Terrestrial Manual for the molecular detection of *B. mallei*.

In addition to *fliP*-IS407A(a), we tested an alternative primer set targeting the same genomic region, referred to in this study as *fliP*-IS407A(b). This set combines the forward primer originally designed by Scholz et al. [16] for endpoint PCR with the reverse primer developed by Tomaso et al. [28] for real-time PCR. This hybrid primer combination has been employed in several studies conducted in Brazil [6,11,12,14,15,31].

Our results revealed a significant discrepancy in performance between the two *fliP*-IS407A primer sets. Notably, the *fliP*-IS407A(b) set yielded the highest number of positive detections, whereas the *fliP*-IS407A(a) set exhibited the lowest sensitivity among the four primer sets evaluated. The difference in detection rates between these two primer sets was statistically significant (Table 3).

Detection failures using the *fliP*-IS407A(a) system have previously been reported in seropositive equids infected with a genetic variant of *B. mallei* in Kuwait. In that study, PCR assays targeting the *B. pseudomallei* complex produced positive results, while the *fliP*-IS407A(a) assay—despite its recommendation by the WOAH—yielded false negatives. However, the presence of *B. mallei* was later confirmed through a species-specific real-time PCR assay and further supported by MLST and SNP genotyping [17]. The authors suggested several possible causes for these false negatives, including mutations at primer or probe binding sites, loss of the IS407A insertion element, or recombination events affecting the targeted genomic region.

The World Organisation for Animal Health [7] has acknowledged that ongoing genetic variation in *B. mallei* may lead to the emergence of strains undetectable by existing PCR protocols. This concern is supported by evidence that the *B. mallei* genome remains dynamic, with frequent recombination events mediated by insertion sequences (ISs) [18,19].

While the reduced performance of the *fliP*-IS407A(a) primer set might be due to the loss of the IS407A insertion site—potentially compromising primer binding—this explanation is complicated by the fact that both the (a) and (b) sets use the same forward primer (Figure 1). Therefore, a complete loss of IS407A would be expected to affect both assays similarly. Furthermore, analysis of seven *B. mallei* genomes indicated that all retained the *fliP* gene, consistently interrupted by the IS407A insertion [18].

Another plausible explanation is the presence of additional insertions that may increase the distance between primers in the *fliP*-IS407A(a) set, potentially leading to failed amplification due to the need for a longer PCR extension time. To investigate this possibility, we analyzed 30 *B. mallei* genome assemblies published within the last decade, selecting only RefSeq-curated entries classified at the scaffold, chromosome, or complete genome level. This dataset included the reference strain ATCC 23344. All genomes were screened for the IS407A insertion sequence and exhibited a conserved structural arrangement. No other mobile genetic elements were identified in proximity to the *fliP* gene, specifically within a 100 base pair window (Table 4).

**Figure 2:**
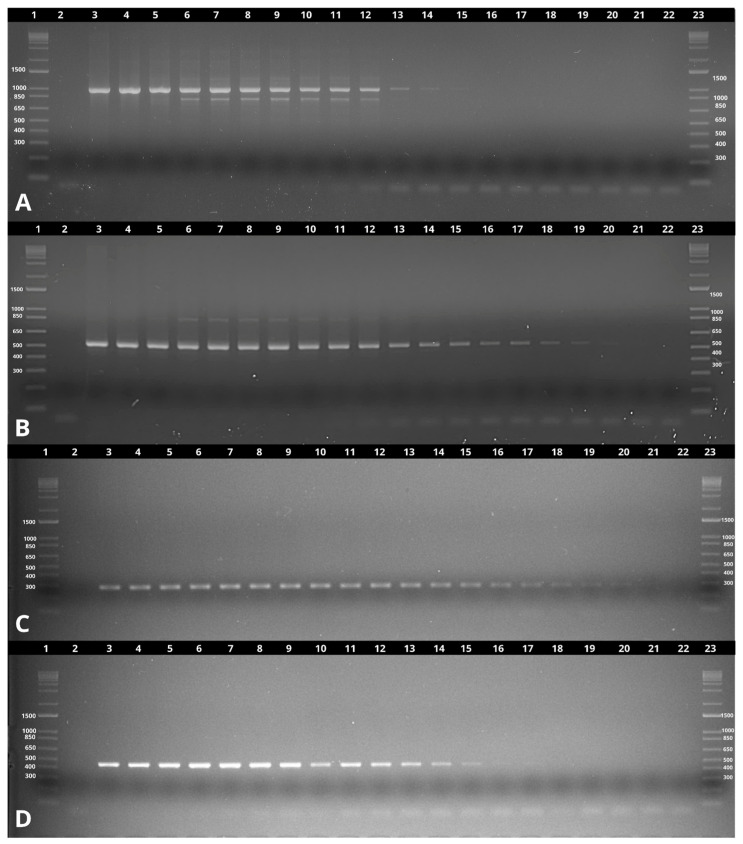
Analytical sensitivity test for each set of primers using the DNA of the *B. mallei* control strain (BAC 86/19). (**A**) fliP-IS407A(a) of *B. mallei* (989 bp). (**B**) fliP-IS407A (b) of *B. mallei* (528 bp). (**C**) Bm17 of *B. mallei* (281 bp). (**D**) Burk457 of *B. mallei* (457 bp). Lane 1: 1 kb plus marker, Lane 2: Negative PCR control, Lane 3: 1000 ng, Lane 4: 500 ng, Lane 5: 250 ng, Lane 6: 125 ng, Lane 7: 62.5 ng, Lane 8: 31.25 ng, Lane 9: 15.625 ng, Lane 10: 7.812 ng, Lane 11: 3.906 ng, Lane 12: 1.953 ng, Lane 13: 0.976 ng, Lane 14: 0.488 ng, Lane 15: 0.244 ng, Lane 16: 0.122 ng, Lane 17: 0.061 ng, Lane 18: 0.030 ng, Lane 19: 0.015 ng, Lane 20: 0.007 ng, Lane 21: 0.003 ng, Lane 22: 0.001 ng, Lane 23: 1 kb plus marker.

In our analytical sensitivity assessment using DNA from the Brazilian *B. mallei* strain BAC 86/19, the *fliP*-IS407A(b) primer set demonstrated superior sensitivity compared to *fliP*-IS407A(a). Specifically, *fliP*-IS407A(a) was able to detect *B. mallei* DNA down to 0.24 ng, whereas *fliP*-IS407A(b) achieved a lower detection limit of 0.007 ng. In contrast, the original study by Scholz et al. [16] reported an analytical sensitivity of 10 fg for the *fliP*-IS407A(a) assay using DNA from *B. mallei* ATCC 23344, corresponding to approximately two genome equivalents. However, we were unable to reproduce this level of sensitivity under our experimental conditions (Figure 2).

The study in which the *fliP*-IS407A(b) primer set was first utilized [11] reports that PCR using *fliP*-IS407A(b) detected a lower PCR threshold (10^1^ CFU/mL) compared to previous studies (10^2^–10^3^ CFU/mL) that standardized PCR for detecting *B. mallei* and *B. pseudomallei* using the FliC and 23S rRNA genes [32].

Taken together, the overall body of evidence suggests that the false-negative results observed with the *fliP*-IS407A(a) PCR are more likely attributable to its lower sensitivity rather than to IS-mediated recombination events.

Differences in sensitivity between primer sets targeting the same genomic region can be attributed to several factors. Primer binding efficiency plays a critical role, as even slight variations in the binding site—due to sequence composition, secondary structures, or GC content—can influence how effectively the primers anneal to the DNA template. Additionally, the design characteristics of the primers themselves, including melting temperature, amplicon length, and susceptibility to forming dimers or hairpins, can significantly affect the overall performance of the assay [33].

The Bm17 primer set was originally developed to amplify a VNTR locus for use in multiple-locus variable-number tandem repeat analysis (MLVA), enabling the genotyping of *B. mallei* and *B. pseudomallei* [30]. Based on NCBI Primer-BLAST analysis (https://www.ncbi.nlm.nih.gov/tools/primer-blast/, accessed on 10 April 2025), this primer set amplifies a 281 bp fragment in *B. mallei* and a 321 bp fragment in *B. pseudomallei*. The 281 bp product was successfully obtained from DNA of a Brazilian *B. mallei* strain isolated from a human patient [15], as well as from clinical equine samples collected in Brazil. Likewise, a 321 bp amplicon was generated from DNA of *B. pseudomallei* isolated from a Brazilian human case of melioidosis [15].

In the analytical sensitivity analysis, the Bm17 primer demonstrated high sensitivity, detecting *B. mallei* DNA at concentrations as low as 0.007 ng. In comparison, the Burk457 primer detected *B. mallei* DNA down to 0.06 ng (Figure 2).

The Burk457 primer set, which targets a hypothetical protein-coding region, was specifically designed for the detection of *B. mallei* DNA and does not amplify DNA from *B. pseudomallei*. During primer standardization, this set successfully detected *B. mallei* directly in tissue samples from 2 out of 10 confirmed equine glanders cases [29].

The comparison of different PCR systems applied to tissue cultures from equids seropositive for glanders revealed marked differences in the performance of the primer sets evaluated. This highlights important diagnostic trade-offs: for example, shorter amplicons such as those targeted by *fliP*-IS407A(b) may enhance sensitivity in samples with degraded or low-concentration DNA, whereas primers targeting VNTR loci like Bm17 provide specificity advantages.

These results underscore the importance of balancing sensitivity and specificity in primer selection, especially in clinical and field testing environments with diverse sample qualities and genetic variants. Employing a combination of primers—such as the highly sensitive *fliP*-IS407A(b) alongside the discriminatory Bm17—may optimize detection accuracy while minimizing false negatives and improving species differentiation.

The data show that selecting primers with higher amplification sensitivity has a direct and significant impact on the detection of *B. mallei*. This variability highlights the critical importance of carefully selecting molecular targets, particularly in samples with low bacterial loads—as is often the case in chronically infected horses—or in situations where pathogen isolation is hindered by the presence of contaminants. The adoption of more sensitive primers can therefore substantially enhance the accuracy of molecular diagnostics under challenging conditions.

## Figures and Tables

**Table 1:** Primers employed in the study.

Target Locus	Sequence (5′ to 3′)	Amplicon Size (pb)	Extension Time (s)	Target Species	Reference
*fliP*-IS407A(a)	F: TCAGGTTTGTATGTCGCTCGG R: CTAGGTGAAGCTCTGCGCGAG	989	60	*B. mallei*	[16]
*fliP*-IS407A(b)	F: TCAGGTTTGTATGTCGCTCGG R: GCCCGACGAGCACCTGATT	528		*B. mallei*	[11]
			60		
Type IV secretion protein Rhs (Burk457)	F: TGTTGACGACGCCTACCATA R: TGCTGCACCTTTGACCTGTA	457	60	*B. mallei*	[29]
VNTR Bm17	F: TATACGCGAGGTTATAACGGATG R: CTTTCTGCTTTTCTAACGTTTCC	281	60	*B. mallei*	[30]
		321		*B. pseudomallei*	

**Table 2:** Percentage of *Burkholderia mallei*-positive microbiological culture samples detected by each PCR primer set individually.

Primers	Total	Positive (%; CI 95%)
fliPIS407A(b)	328	282 (86.0; 81.7–89.5)
Bm17	328	250 (76.2; 71.2–80.7)
Burk457	328	115 (35.1; 30.0–40.5)
fliP_IS407A(a)	328	44 (13.4; 10.0–17.7)

**Table 3:** Performance of PCR primer sets in detecting *Burkholderia mallei* DNA in all microbiological culture samples.

Specimens	Total	Query Method	Standard Method	P-P	N-N	P-N	N-P	Positive (%)	*p*-Values	K	Sens (%)	Spec (%)	PPV (%)	NPV (%)
All	328	Bm17	*fliP*-IS407A(b)	207	3	43	75	325 (99.08)	0.004124	−0.15	73.40	6.52	82.8	3.84
All	328	Burk457	*fliP*-IS407A(b)	108	39	7	174	289 (88.10)	<0.000001	0.09	38.30	84.78	93.91	18.31
All	328	*fliP*-IS407A(b)	*fliP*-IS407A(a)	43	45	239	1	283 (86.28)	<0.000001	0.04	97.73	15.85	15.25	97.83
All	328	Bm17	Burk457	105	68	10	145	260 (79.26)	<0.000001	18	42.00	87.18	91.30	31.92
All	328	Bm17	*fliP*-IS407A(a)	42	76	208	2	252 (76.82)	<0.000001	0.07	95.45	26.76	16.8	97.44
All	328	Burk457	*fliP*-IS407A(a)	35	204	80	9	124 (37.80)	<0.000001	0.30	79.55	71.83	30.43	95.77

P-P: positive/positive; N-N: negative/negative; P-N: positive/negative; N-P: negative/positive; Positive: positive in both methods or at least in one of the two; K: Cohen’s Kappa; Sens: sensitivity; Spec: specificity; PPV: positive predictive value; NPV: negative predictive value.

**Table 4:** Structural analysis of the *fliP* gene region and presence of IS407A in 30 *Burkholderia mallei* genomes.

*Burkholderia mallei* Genome	Start Position of *fliP*	End Position of *fliP*	Start Position of IS407A	End Position of IS407A	Distance Between *fliP* and IS407A	Biotype	Other Insertion Sequences (Besides IS407A) *
GCF_000959165.1.gtf	84,407	84,958	85,009	85,868	51	Pseudogene	Absent
GCF_000959405.1.gtf	800,547	801,098	801,149	802,008	51	Pseudogene	Absent
GCF_000959465.1.gtf	826,052	826,603	826,654	827,513	51	Pseudogene	Absent
GCF_000959485.1.gtf	1,406,667	1,407,218	1,407,270	1,408,129	52	Pseudogene	Absent
GCF_000959585.1.gtf	874,920	875,471	874,010	874,869	51	Pseudogene	Absent
GCF_000959625.1.gtf	385,994	386,545	386,596	387,455	51	Pseudogene	Absent
GCF_001279165.1.gtf	2,956,800	2,957,351	2,955,890	2,956,749	51	Pseudogene	Absent
GCF_001279265.1.gtf	2,878,722	2,879,273	2,877,812	287,8671	51	Pseudogene	Absent
GCF_001729545.1.gtf	121,007	121,558	120,097	120,956	51	Pseudogene	Absent
GCF_002345985.1.gtf	547,476	548,027	546,566	547,425	51	Pseudogene	Absent
GCF_002346005.1.gtf	547,470	548,021	546,560	547,419	51	Pseudogene	Absent
GCF_002346025.1.gtf	547,476	548,027	546,566	547,425	51	Pseudogene	Absent
GCF_002346045.1.gtf	2,785,740	2,786,291	2,786,342	2,787,201	51	Pseudogene	Absent
GCF_002346065.1.gtf	547,491	548,042	546,581	547,440	51	Pseudogene	Absent
GCF_002346085.1.gtf	547,484	548,035	546,574	547,433	51	Pseudogene	Absent
GCF_002346105.1.gtf	547,490	548,041	546,580	547,439	51	Pseudogene	Absent
GCF_002346125.1.gtf	547,469	548,020	546,559	547,418	51	Pseudogene	Absent
GCF_002346145.1.gtf	547,478	548,029	546,568	547,427	51	Pseudogene	Absent
GCF_002346165.1.gtf	547,477	548,028	546,567	547,426	51	Pseudogene	Absent
GCF_002346185.1.gtf	547,457	548,008	546,547	547,406	51	Pseudogene	Absent
GCF_002346205.1.gtf	2,785,656	2,786,207	2,786,258	2,787,117	51	Pseudogene	Absent
GCF_003933015.1.gtf	2,114,566	2,115,117	2,115,168	2,116,027	51	Pseudogene	Absent
GCF_003933025.1.gtf	3,349,478	3,350,029	3,348,568	3,349,427	51	Pseudogene	Absent
GCF_003933035.1.gtf	129,161	129,712	128,251	129,110	51	Pseudogene	Absent
GCF_003933045.1.gtf	1,356,884	1,357,435	1,355,974	1,356,833	51	Pseudogene	Absent
GCF_033870355.1.gtf	872,844	873,395	871,934	872,793	51	Pseudogene	Absent
GCF_033870375.1.gtf	873,825	874,376	872,915	873,774	51	Pseudogene	Absent
GCF_033870395.1.gtf	3,142,218	3,142,769	3,141,308	3,142,167	51	Pseudogene	Absent
GCF_033956065.1.gtf	2,785,836	2,786,387	278,6438	2,787,297	51	Pseudogene	Absent
GCF_939576165.1.gtf	3,260,392	3,260,943	3,260,994	3,261,853	51	Pseudogene	Absent

* Within a 100 base pair window.

## Data Availability

The datasets used and/or analyzed during the current study are available from the corresponding author on reasonable request.

## References

[1] Kettle A.N., Wernery U. (2016). Glanders and the risk for its introduction through the international movement of horses. Equine Vet. J..

[2] Coleman W., Ewing J. (1903). A Case of Septicemic Glanders in the Human Subject. J. Med. Res..

[3] Pilcher J.T. (1907). Glanders in the human subject: Clinical report of two cases observed in the fourth medical division of bellevue hospital of new york. Ann. Surg..

[4] Lowe W., March J.K., Bunnell A.J., O’Neill K.L., Robison R.A. (2014). PCR-based Methodologies Used to Detect and Differentiate the *Burkholderia pseudomallei* complex: *B. pseudomallei*, *B. mallei*, and *B. thailandensis*. Curr. Issues Mol. Biol..

[5] Wittig M.B., Wohlsein P., Hagen R.M., Al Dahouk S., Tomaso H., Scholz H.C., Nikolaou K., Wernery R., Wernery U., Kinne J. (2006). Ein Ubersichtsreferat zur Rotzerkrankung [Glanders—A comprehensive review]. Dtsch. Tierarztl. Wochenschr..

[6] Suniga P.A.P., Mantovani C., Dos Santos M.G., do Egito A.A., Verbisck N.V., Dos Santos L.R., Dávila A.M.R., Zimpel C.K., Zerpa M.C.S., Chiebao D.P. (2023). Glanders Diagnosis in an Asymptomatic Mare from Brazil: Insights from Serology, Microbiological Culture, Mass Spectrometry, and Genome Sequencing. Pathogens.

[7] WOAH, World Organisation for Animal Health (2024). Chapter 3.6.11 Glanders and Mielioidosis. Manual of Diagnostic Tests and Vaccines. https://www.woah.org/fileadmin/Home/eng/Health_standards/tahm/3.06.11_GLANDERS.pdf.

[8] Mota R.A., Brito M.F., Castro F.J., Massa M. (2000). Mormo em eqüídeos nos estados de Pernambuco e Alagoas. Pesqui. Vet. Bras..

[9] Mota R.A., Oliveira A.A.D.F., Pinheiro Junior J.W., Silva L.B.G.D., Brito M.D.F., Rabelo S.S.A. (2010). Glanders in donkeys (*Equus asinus*) in the state of Pernambuco, Brazil: A case report. Braz. J. Microbiol..

[10] Rocha L.O.D., Lima L.A.R.D., Albuquerque R.M.S.D., Lages S.L.S., Nunes A.C.B.T., Castro R.S.D., Falcão M.V.D. (2021). Monitoring the outbreak of equine glanders in Alagoas, Brazil: Clinical, immunological, molecular and anatomopathological findings. Ciênc. Rural..

[11] Abreu D.C., Gomes A.S., Tessler D.K., Chiebao D.P., Fava C.D., Romaldini A.H.C.N., Araujo M.C., Pompei J., Marques G.F., Harakava R. (2020). Systematic monitoring of glanders-infected horses by complement fixation test, bacterial isolation, and PCR. Vet. Anim. Sci..

[12] Suniga P.A.P., Mantovani C., Santos M.G., Rieger J.S.G., Gaspar E.B., Dos Santos F.L., Mota R.A., Chaves K.P., Egito A.A., Filho J.C.O. (2023). Molecular detection of *Burkholderia mallei* in different geographic regions of Brazil. Braz. J. Microbiol..

[13] Rocha L.S., Oliveira A.L.F., Arruda F.P., Pitchenin L.C., Dutra V., Nakazato L., Furlan F.H., Colodel E.M. (2023). Pathology, microbiology, and molecular evaluation of tissues from equids serologically positive for *Burkholderia mallei* in Midwestern Brazil. Pesqui. Veter-Bras..

[14] Nassar A.F.C., Chiebao D.P., Fava C.D., Miyashiro S., Castro V., Ogata R.A., Yamamora J.M., Monteiro C.A.S., Monteiro E.J.B. (2025). Histopathological and diagnostic aspects of glanders based on a case series from Brazil. J. Equine Vet. Sci..

[15] Luz K.G., Bezerra F.R.O., Sicolo M.A., Silva A.A.R.S., Egito A.A., Suniga P.A.P., Moriya J.C.K., Santos M.G., Mantovani C., Silva J.S. (2024). Clinical and Molecular Characterization of Human *Burkholderia mallei* Infection, Brazil. Emerg. Infect. Dis..

[16] Scholz H.C., Joseph M., Tomaso H., Al Dahouk S., Witte A., Kinne J., Hagen R.M., Wernery R., Wernery U., Neubauer H. (2006). Detection of the reemerging agent *Burkholderia mallei* in a recent outbreak of glanders in the United Arab Emirates by a newly developed fliP-based polymerase chain reaction assay. Diagn. Microbiol. Infect. Dis..

[17] Laroucau K., Aaziz R., Vorimore F., Varghese K., Deshayes T., Bertin C., Delannoy S., Sami A.M., Al Batel M., El Shorbagy M. (2021). A genetic variant of *Burkholderia mallei* detected in Kuwait: Consequences for the PCR diagnosis of glanders. Transbound Emerg Dis..

[18] Losada L., Ronning C.M., DeShazer D., Woods D., Fedorova N., Kim H.S., Shabalina S.A., Pearson T.R., Brinkac L., Tan P. (2010). Continuing evolution of *Burkholderia mallei* through genome reduction and large-scale rearrangements. Genome Biol. Evol..

[19] Song H., Hwang J., Yi H., Ulrich R.L., Yu Y., Nierman W.C., Kim H.S. (2010). The early stage of bacterial genome-reductive evolution in the host. PLoS Pathog..

[20] MAPA (2018). Portaria SDA n° 35 de 17 de Abril de 2018. https://www.gov.br/agricultura/pt-br/assuntos/lfda/legislacao-metodos-da-rede-lfda/copy_of_diagnostico-animal%20arquivos/copy_of_Portaria35de17.04.2018Testeslaboratparamormo.pdf/view.

[21] Sauer S., Freiwald A., Maier T., Kube M., Reinhardt R., Kostrzewa M., Geider K. (2008). Classification and identification of bacteria by mass spectrometry and computational analysis. PLoS ONE.

[22] Freiwald A., Sauer S. (2009). Phylogenetic classification and identification of bacteria by mass spectrometry. Nat. Protoc..

[23] Bacanelli G., Olarte L.C., Silva M.R., Rodrigues R.A., Carneiro P.A., Kaneene J.B., Verbisck N.V. (2019). Matrix assisted laser desorption ionization-time-of-flight mass spectrometry identification of *Mycobacterium bovis* in Bovinae. J. Vet. Med. Sci..

[24] Lasch P., Beyer W., Bosch A., Borriss R., Drevinek M., Dupke S., Ehling-Schulz M., Gao X., Grunow R., Jacob D. (2025). A MALDI-ToF mass spectrometry database for identification and classification of highly pathogenic bacteria. Sci Data..

[25] Verbisck N.V., de Araújo F.R., Gaspar E.B., Júnior A.A.F., Mota R.A., da Silva K.P.C., Lima D.A.R. (2020). Caracterização e Identificação de Burkholderia Mallei por Espectrometria de Massas MALDI-TOF: Resultados de um Estudo Piloto.

[26] Kinoshita Y., Cloutier A.K., Rozak D.A., Khan M.S.R., Niwa H., Uchida-Fujii E., Katayama Y., Tuanyok A. (2019). A novel selective medium for the isolation of *Burkholderia mallei* from equine specimens. BMC Vet. Res..

[27] van Embden J.D., Cave M.D., Crawford J.T., Dale J.W., Eisenach K.D., Gicquel B., Hermans P., Martin C., McAdam R., Shinnick T.M. (1993). Strain identification of *Mycobacterium tuberculosis* by DNA fingerprinting: Recommendations for a standardized methodology. J. Clin. Microbiol..

[28] Tomaso H., Scholz H.C., Al Dahouk S., Eickhoff M., Treu T.M., Wernery R., Wernery U., Neubauer H. (2006). Development of a 5′-nuclease real-time PCR assay targeting fliP for the rapid identification of *Burkholderia mallei* in clinical samples. Clin. Chem..

[29] Fonseca Júnior A.A., Pinto C.A., Alencar C.A.S., Bueno B.L., Dos Reis J.K.P., de Carvalho Filho M.B. (2021). Validation of three qPCR for the detection of *Burkholderia mallei* in equine tissue samples. Arch. Microbiol..

[30] Yen M.W.S., Lisanti O., Thibault F., San T.S., Kee L.G., Hilaire V., Jiali L., Neubauer H., Vergnaud G., Ramisse V. (2009). Validation of ten new polymorphic tandem repeat loci and application to the MLVA typing of *Burkholderia pseudomallei* isolates collected in Singapore from 1988 to 2004. J. Microbiol. Methods.

[31] Gaspar E.B., Santos L.R.D., Egito A.A.D., Santos M.G.D., Mantovani C., Rieger J.D.S.G., Abrantes G.A.S., Suniga P.A.P., Favacho J.M., Pinto I.B. (2023). Assessment of the Virulence of the *Burkholderia mallei* Strain BAC 86/19 in BALB/c Mice. Microorganisms.

[32] Altukhova V.V., Antonov V.A., Tkachenko O.V., Zinchenko O.V., Zamarano V.S., Plekhanova N.G., Ilyukhen V.I., Torfimov D.Y. (2007). Use of the polymerase chain reaction to detect the glanders and melioidosis pathogen in experimental infection. Mol. Genet. Microbiol. Virol..

[33] Bustin S., Huggett J. (2017). qPCR primer design revisited. Biomol. Detect. Quantif..

